# Neurological Implications of Vitamin B12 Deficiency in Diet: A Systematic Review and Meta-Analysis

**DOI:** 10.3390/healthcare11070958

**Published:** 2023-03-27

**Authors:** Mubarak Alruwaili, Rehana Basri, Raed AlRuwaili, Anas Mohammad Albarrak, Naif H. Ali

**Affiliations:** 1 Department of Internal Medicine, College of Medicine, Jouf University, Sakaka 72345, Saudi Arabia; 2 Department of Internal Medicine, College of Medicine, Prince Sattam Bin Abdulaziz University, Alkharj 11942, Saudi Arabia; 3 Department of Internal Medicine, Medical College, Najran University, Najran 55461, Saudi Arabia

**Keywords:** cognition, dietary patterns, neurology, nutritional profile, Vitamin B12

## Abstract

Background: Vitamin B12 is one of the most important B-Vitamins that the human body needs on a daily basis, the lack of which can precipitate several neurological issues. Objectives: This systematic aimed to investigate the neurological implications of Vitamin B12 deficiency and the effects when B12 levels were corrected in susceptible individuals. Methods: The databases PubMed-MEDLINE, Web of Science, Cochrane, and Scopus were all searched using pertinent keywords, reference searches, and citation searches. The terms used to access the database were “Cognition”, “Dietary patterns”, “Neurology”, “Nutritional profile”, and “Vitamin B12”. Results: Vitamin B12 was shown to noticeably improve cognition and other neurological parameters in the short term in older adults and the short-to-medium term in children; however, there was no perceived increase/improvement when the Vitamin was administered in the longer term, either alone or in conjunction with other similar nutritional interventions. Conclusion: Vitamin B12’s role in the improvement of neurological functions over a long-term period remains somewhat inconclusive to date, as the majority of our selected control trials did not display much correlation between the two factors. However, Vitamin B12 did improve cognition levels in both children and older adults over a short course of administration.

## 1. Introduction

The sophisticated molecular molecule called B12 is water soluble. It is essential for the normal development of people, animals, and even a few microorganisms [1]. It must be obtained through food because the human body is unable to produce enough of it. It has a complex structure and a metallic particle in addition to cobalt. It comes in a variety of forms. However, cobalamin and cyano cobalamin are the two most common forms [2]. It is created by microorganisms in livestock and cows. In cows, it travels from the rumen and other organs to the muscle. Humans ingest cow’s meat, which contains this Vitamin [3]. Dairy goods and eggs are extra nutrient-rich foods. Strict vegetarians who experience B12 deficiency must take supplements enriched with B12 [4]. B12 can be eliminated from the body because it is water soluble. Furthermore, it cannot be stored in fatty acids or adipose cells. A severe B12 deficiency has been linked in some cases [5,6] to neuropathy, pernicious anaemia, ileal resections, and other gastrointestinal complications. Pernicious anaemia is rarely reported in people who are dietary B12 deficient, with the exception of small infants who are solely breastfed or severe vegetarians [5,7].

Other kinds of B12 include methylcobalamin, deoxyadenosylcobalamin, hydroxocobalamin, and cyanocobalamin. Methyl cobalamin, which is also present in dietary supplements, is the most active form of cobalamin discovered in human circulation. This form must be converted by the organism into either methyl cobalamin or 5-deoxyandenosylcobalamin in order to be absorbed [8]. Cobalamin is taken through the ileum’s cubilin receptor. Its complex structure is made up of megalin, cubilin, and amnion-related transmembrane protein (AMN) [9]. Its 460 kDa molecular weight and location in the proximal tubule make it interesting. At a pH of 5.4, which is acidic, absorption happens. Three cobalamin-binding proteins (ascobalo-philins), one carrier protein (haptocorrin), and the R protein are associated with absorption in adult granulocytes and monocytes of progenitor cells. Additionally, it has been discovered in exocrine epithelial cells that secrete saliva, bile, gastric acid, and breast milk [9].

People of all ages, financial statuses, races, and sexes are susceptible to deficiency. It is the most common dietary deficiency in the United States [1]. Early identification and therapy are essential to prevent neurological issues, poor outcomes, and premature mortality [10]. Pernicious anaemia is the most common source of B12 deficiency. An intrinsic factor required for gastrointestinal absorption of Vitamin B12 from the diet is absent in this auto-immune disease. However, women, people over 60, and those who have auto-immune illnesses such as Addison’s disease and vitiligo are more likely to have the disorder [11]. The illness known as autoimmune gastrectomy (AG) happens when the body produces antibodies against healthy stomach cells that are usually produced against viruses and bacteria. Healthy stomach cells that generate acidic fluids are impacted by autoimmune AG. The intrinsic component that causes pernicious anaemia is also affected by these antibodies.

These days, people of all ages have psychiatric problems, acute anxiety, and sadness. Although these people are given expensive psychiatric medications, opioids, or benzodiazepines, in many instances, there is also the presence of an underlying moderate-to-severe level of Vitamin B12 deficiency [3]. Hence, through this systematic review and meta-analysis, we aimed to shed light upon the neurological implications of diet in cases in which the individual is suffering from a Vitamin B12 deficiency, and if the introduction of this Vitamin into the diet can help to ameliorate some of the symptoms that accompany the neurological ailment. We aimed to analyze the effect of Vitamin B12 in the human diet and its correlation with the neurological health of an individual.

## 2. Materials and Methods

### 2.1. Protocol Employed

**Figure 1:**
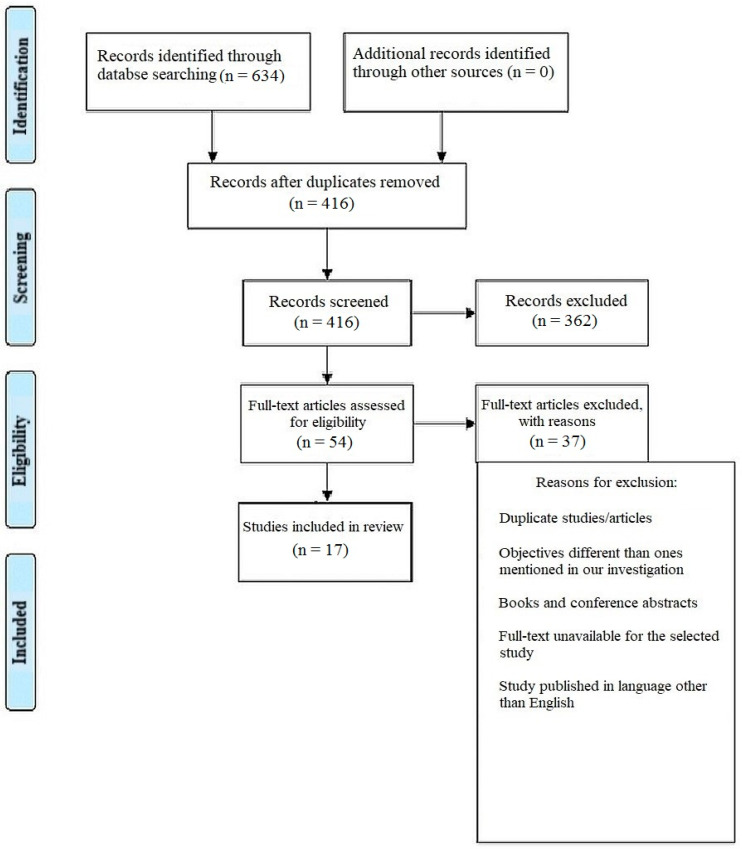
Representation of selection of articles through PRISMA framework.

This systematic review was performed as per the Preferred Reporting Items for Systematic Review and Meta-analysis (PRISMA) (Figure 1) strategy and rules from the Cochrane group, and using the book *Orderly Reviews in Healthcare: Meta-Examination* [12], with the PROSPERO registration number being CRD42023387063.

### 2.2. Review Hypotheses

Through this systematic review, our primary objective was to review studies that were published in the neurological literature and discussed the effects of Vitamin B12 deficiency on the neurological parameters of individuals, such as cognition, overall health and/or any pre-existing neurological/systemic disorder.

### 2.3. Study Selection Process

There were a total of 634 documents discovered after extensive search of the online journals, and 416 of the papers were selected initially. Following that, 362 similar/duplicate articles were eliminated, which resulted in 54 separate papers being available. The abstracts and titles of submissions were then reviewed, and a further 37 papers were eliminated. Finally, 17 documents that met the requisite inclusion and exclusion criteria were chosen; these primarily included in vitro studies, literature reviews and comparative assessments.

### 2.4. Inclusion Criterion

Articles that contained relevant data for our review objectives were selected for full-text screening. Studies that reported clinical trials, in vitro studies, randomised/non-randomised studies, systematic/literature reviews containing substantial sample volume and detailed case reports were considered for inclusion in our review. We also monitored studies that possessed higher methodological quality.

### 2.5. Exclusion Criteria

The following were excluded from the scope of our systematic review: incomplete data, seminar presentations, scholarly articles, placebo-controlled studies, and opinion articles.

Since the literature available on this topic was quite scant in volume, we did not limit our search in terms of the time period in which the studies were published, i.e., we took into account all the papers that were published within the context of our topic (where the number of papers itself was found to be quite sparse in number). Additionally, literature reviews and cases published in languages other than English were excluded.

### 2.6. Search Strategy

Using relevant keywords, reference searches, and citation searches, the databases PubMed-MEDLINE, Web of Science, Cochrane, and Scopus were all searched. “Cognition”, “Dietary patterns”, “Neurology”, “Nutritional profile” and “Vitamin B12” were the search terms used to access the database.

### 2.7. Data Selection and Coding

Two independent reviewers located the relevant papers by using the right keywords in various databases and online search tools. The chosen articles were compared, and a third reviewer was brought in if there was a dispute.

After choosing the articles, the same two reviewers independently extracted the following data: author, year of publication, country, kind of publication, study topic, population demographics (n, age), outcome measure(s), relevant result(s), and conclusion(s). The data were compared and any differences were discussed with the third reviewer.

### 2.8. Risk of Bias Assessment

The AMSTAR-2 technique [13] was used to evaluate the risk of bias in the studies we chose (Table 1). AMSTAR 2 joins a number of other instruments that have been released for this purpose, as a critical evaluation tool for systematic reviews. As seen in Table 2 below, it is a 16-point checklist. Two instruments that have drawn a lot of attention served as the foundation for the creation of the original AMSTAR tool. The original AMSTAR was duplicated in two newly produced instruments. The AMSTAR items identify the domains specified in the Cochrane risk of bias instruments for systematic reviews. In each case, these indicate an agreement that was achieved after input from more than 30 methodology experts.

### 2.9. Statistical Analysis

**Figure 2:**
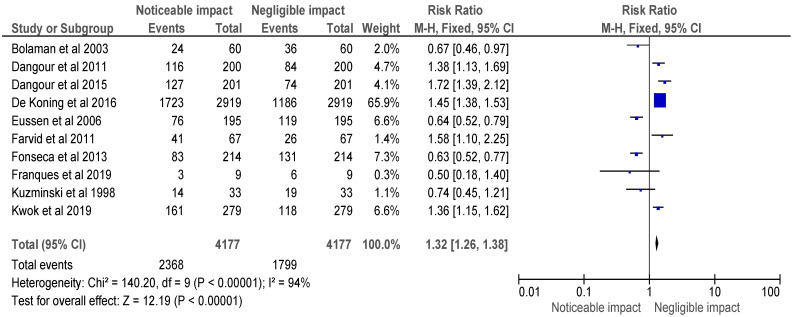
Risk ratio of selected randomised control trials represented on a forest plot after their meta-analysis, in which the noticeable impact of Vitamin B12 interventions compared to their negative to negligible impact was assessed (where the blue dot represents the study with the most weight and the rhombus represents the total weight of the forest plot) [15,16,17,18,19,20,21,22,26,27].

**Figure 3:**
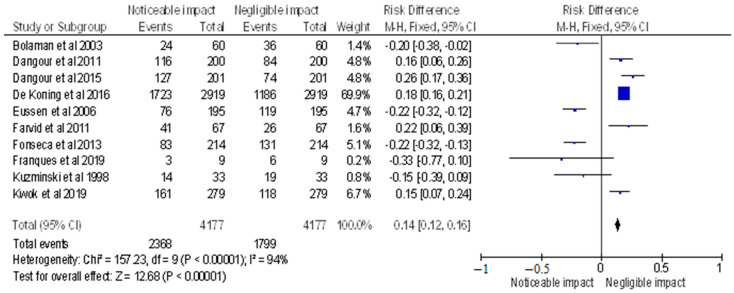
Risk difference of selected randomised control trials represented on a forest plot after their meta-analysis, in which the noticeable impact of Vitamin B12 interventions compared to their negative to negligible impact was assessed (where the blue dot represents the study with the most weight and the rhombus represents the total weight of the forest plot) [15,16,17,18,19,20,21,22,26,27].

**Figure 4:**
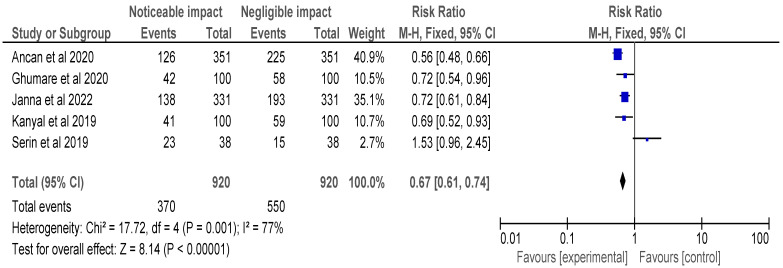
Risk ratio of selected cross-sectional, cohort and retrospective investigations represented on a forest plot after their meta-analysis, in which the noticeable impact of Vitamin B12 interventions compared to their negative to negligible impact was assessed (where the blue dot represents the study with the most weight and the rhombus represents the total weight of the forest plot) [14,23,24,25,30].

**Figure 5:**
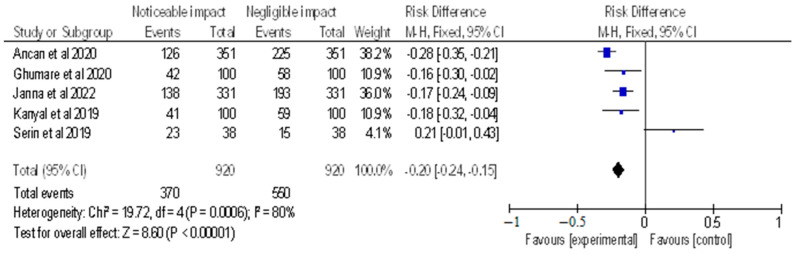
Risk difference of selected cross-sectional, cohort and retrospective investigations represented on a forest plot after their meta-analysis, in which the noticeable impact of Vitamin B12 interventions compared to their negative to negligible impact was assessed (where the blue dot represents the study with the most weight and the rhombus represents the total weight of the forest plot) [14,23,24,25,30].

After selecting data on the sample size, variables analyzed, and various elements of the investigations, the data were then entered into the Revman 5 programme for meta-analysis. Forest plots illustrating the risk ratio (RR), and risk difference (RD) for different study methodologies were obtained as part of the meta-analysis for our study, as shown in Figure 2, Figure 3, Figure 4 and Figure 5.

## 3. Results

The AMSTAR-2 [14] checklist is displayed in Table 1. The study design, methodology employed, description and outcome are mentioned in Table 2. The results of the meta-analysis are provided in Figure 2, Figure 3, Figure 4 and Figure 5.

The meta-analysis of the included selected randomised control trials is represented in Figure 2, and a forest plot was used to represent the RR of the studies, which was found to be 1.32 [1.26, 1.38]. The analysis revealed a noticeable impact of Vitamin B12 interventions, as compared to its negative to negligible impact. Heterogeneity was evaluated using Chi^2^ = 140.20, df = 9 (*p* < 0.00001); I^2^ = 94%, indicating significant heterogeneity among the studies. The test for overall effect was conducted using Z = 12.19 (*p* < 0.00001), indicating a statistically significant effect of Vitamin B12 interventions on neurological outcomes. These findings suggest that Vitamin B12 supplementation may have a beneficial impact on neurological function in individuals with a deficiency. However, further research is needed to determine the optimal dosing and duration of Vitamin B12 interventions in this population.

The meta-analysis included selected randomised control trials, and the forest plot depicted the RD of the studies (displayed in Figure 3), which was found to be 0.14 [0.12, 0.16]. The analysis revealed a noticeable impact of Vitamin B12 interventions, as compared to its negative to negligible impact. Heterogeneity was assessed using Chi^2^ = 157.23, df = 9 (*p* < 0.00001); I^2^ = 94%, indicating significant heterogeneity among the studies. The test for overall effect was conducted using Z = 12.68 (*p* < 0.00001), indicating a statistically significant effect of Vitamin B12 interventions on neurological outcomes. These findings suggest that Vitamin B12 supplementation may have a positive impact on neurological function in individuals with a deficiency. However, further research is required to determine the optimal dosage and duration of Vitamin B12 interventions in this population. The results of this meta-analysis provide evidence for the importance of adequate Vitamin B12 intake in maintaining neurological health.

Figure 4’s meta-analysis included selected cross-sectional, cohort, and retrospective investigations and the forest plot depicted the RR of the studies, which was found to be 0.67 [0.61, 0.74]. The analysis revealed a noticeable impact of Vitamin B12 interventions, as compared to their negative to negligible impact. Heterogeneity was assessed using Chi^2^ = 17.72, df = 4 (*p* = 0.001); I^2^ = 77%, indicating significant heterogeneity among the studies. The test for overall effect was conducted using Z = 8.14 (*p* < 0.00001), indicating a statistically significant effect of Vitamin B12 interventions on neurological outcomes. These findings suggest that Vitamin B12 supplementation may have a protective effect on neurological function in individuals with a deficiency. However, further research is needed to determine the optimal dosing and duration of Vitamin B12 interventions in this population. It is important to note that the studies included in this meta-analysis were observational in nature, and therefore, causality cannot be established. Overall, the results of this meta-analysis provide support for the importance of adequate Vitamin B12 intake in maintaining neurological health.

The meta-analysis (as shown in Figure 5) included selected cross-sectional, cohort, and retrospective investigations, and the forest plot depicted the RD of the studies, which was found to be −0.20 [−0.24, −0.15]. The analysis revealed a noticeable impact of Vitamin B12 interventions, as compared to their negative to negligible impact. Heterogeneity was assessed using Chi^2^ = 19.72, df = 4 (*p* = 0.0006); I^2^ = 80%, indicating significant heterogeneity among the studies. The test for overall effect was conducted using Z = 8.60 (*p* < 0.00001), indicating the statistically significant effect of Vitamin B12 interventions on neurological outcomes. These findings suggest that Vitamin B12 supplementation may have a beneficial impact on neurological function in individuals with a deficiency. However, further research is needed to determine the optimal dosing and duration of Vitamin B12 interventions in this population. The results of this meta-analysis provide evidence for the importance of adequate Vitamin B12 intake in maintaining neurological health. It is important to note that the studies included in this meta-analysis were observational in nature, and therefore, causality cannot be established.

## 4. Discussion

This systematic review and meta-analysis included a total of 17 studies and aimed to assess the neurological implications of Vitamin B12 deficiency in diet. The meta-analysis included selected randomised control trials and observational studies, which were analyzed using appropriate statistical methods.

The findings of the meta-analysis revealed a noticeable impact of Vitamin B12 interventions, as compared to its negative to negligible impact, on neurological outcomes in individuals with a deficiency. The meta-analysis showed that the risk ratio and risk difference for selected randomised control trials were 1.32 [1.26, 1.38] and 0.14 [0.12, 0.16], respectively, while for selected cross-sectional, cohort, and retrospective investigations, they were −0.20 [−0.24, −0.15] and 0.67 [0.61, 0.74], respectively. The heterogeneity across the studies was also assessed and found to be significant.

The significant heterogeneity in the studies suggests that the optimal dosing and duration of Vitamin B12 interventions in individuals with a deficiency require further investigation. However, the findings of this meta-analysis highlight the importance of adequate Vitamin B12 intake in the diet for maintaining neurological health. In summary, this systematic review and meta-analysis provide important insights into the role of Vitamin B12 in maintaining neurological health, particularly in individuals with a deficiency. The findings underscore the need for healthcare providers to ensure that individuals with a risk of Vitamin B12 deficiency receive appropriate dietary counselling and supplementation to prevent or alleviate neurological symptoms associated with a deficiency. The results of this study have important implications for public health policy and clinical practice, and provide valuable information for future research in this area.

Adults between the ages of 40 and 90 are more likely than younger adults to experience psychiatric issues as a result of B12 deficiency [31]. Cognitive changes such as memory loss, depression, delusions, hallucinations, and dementia are some of the psychiatric manifestations [32]. The causes include unstable neurotransmitter production, high homocysteine levels and elevated levels of methylmalonic acid (MMA) in B12 deficient individuals. If there is no other obvious cause of a psychiatric disorder, screening and B12 supplementation should be taken into consideration. The development of the foetus’ brain also depends critically on Vitamin B12 and folate. In addition, both are essential for infants’ myelination during the first two years of life and until puberty [33]. Depending on the part of the nervous system that is affected by B12 deficiency, a child may develop a variety of cognitive and intellectual issues. In order to prevent neurological disorders in the developing foetus, pregnant women who are deficient in both need to take supplements. Since elderly people have trouble absorbing this Vitamin from food sources, supplements can be used to make up for any deficiencies [34]. Supplements may be used to restore deficiencies that vegans may experience.

Older people frequently lack Vitamin B12, primarily due to malabsorption [24]. Cognitive impairment is linked to a high prevalence of Vitamin B12 status impairment. Such correlations might not, however, be causal. Additionally, although the estimated percentage of real reversible dementia in people with Vitamin B12 insufficiency is low [35], poor Vitamin B12 metabolism may be one of several variables that contribute to the onset of cognitive impairment and dementia and affect the disease’s course. Unknown factors that may contribute to this phenomenon include decreased methylation capacity, increased homocysteine concentrations and B Vitamins’ role in maintaining the blood–brain barrier’s integrity [36].

Since norms for cognitive performance [37] are rarely population specific and there are no age-specific reference data for neurologic function in older adults [38], interpretation is difficult. It is possible that study participants’ neurologic and cognitive function was not compromised at the start of the investigation. However, regardless of baseline function, the goal of our study was to find any neurologic and cognitive benefits of Vitamin B12 supplementation in older persons with moderate Vitamin B12 deficiency. The trial participants in our review were generally healthy, so it is possible that the findings do not apply to the entirety of the elderly population. Additionally, the course of treatment might have been too brief, and any effects of Vitamin B12 supplementation might not have been seen until years later or during follow-up [39]. To our knowledge, no relevant experiment using Vitamin B12 supplementation for a duration longer than two years has been carried out [39]. Direct links between nerve conduction and Vitamin B12 levels have been observed in observational studies [40], although this finding has not always been the case [40,41]. No prior randomised controlled trial has, to our knowledge, examined the effect of Vitamin B12 supplementation on neurologic function in elderly individuals. Prior studies examining the impact of Vitamin B12 supplementation on cognitive performance mostly failed to demonstrate any positive effects, although they were of inconsistent quality, small scale, and short duration [39,42]. Recently, there has been some indication of the benefit of taking numerous B Vitamins, particularly in subgroups of people with worse biochemical state when randomly assigned at baseline [43]. Our systematic study adds solid information on the impact of Vitamin B12 on cognitive performance as people age, and the results are in line with a recent meta-analysis that found no association between Vitamin B12 intake and cognitive ageing [44].

Our study included a higher percentage of randomised clinical trials, which could be said to be a major drawback, alongside the fact that the overall number of investigations that we selected for assessment and subsequent meta-analysis might be deemed to be less than ideal. The lack of studies assessing the long-term effects (>2 years for example) of Vitamin B12 on the neurological parameters of individuals can be identified as another limitation of this review, but the fact that no such current investigation could be found in the literature warrants further research in this field so that a unified consensus can be reached with respect to the effect of Vitamin B12 in alleviating neurological decline in susceptible individuals.

Despite the valuable insights provided by this systematic review and meta-analysis, there are some limitations to consider. Firstly, the studies included in the meta-analysis were heterogeneous in terms of study design, participant characteristics, dosages, and the duration of Vitamin B12 interventions. Secondly, the studies included were conducted in different populations with varying risk factors for Vitamin B12 deficiency. This could have introduced bias into the findings of the meta-analysis. Thirdly, the studies were conducted in different countries and settings, which could have influenced the results due to variations in dietary and lifestyle factors. Fourthly, the quality of the studies included in the meta-analysis varied, which could have affected the robustness of the findings. Lastly, the meta-analysis did not assess potential publication bias, which could have influenced the findings. Therefore, caution should be taken when interpreting the results of this meta-analysis, and further research is needed to address these limitations and provide more conclusive evidence regarding the role of Vitamin B12 in neurological health.

## 5. Conclusions

Through this systematic review and subsequent meta-analysis, it was determined that the majority of the studies that were chosen did not find any advantages of daily Vitamin B12 supplementation over a long period of time (around 1 year or more) on neurologic or cognitive function in older people with moderate Vitamin B12 deficiency who were asymptomatic, nonanemic, and without anaemia. These findings are directly applicable to modern clinical practice, which has identified low Vitamin B12 status as a risk factor for neurologic and cognitive impairments, particularly in older adults. Further, the current study’s findings raise concerns about the usefulness of screening for mild Vitamin B12 deficiency in the absence of anaemia and signs of neurologic or cognitive impairment, and point to the need for stricter definitions of Vitamin B12 deficiency.

## Figures and Tables

**Table 1:** AMSTAR-2 16-point checklist of risk of bias assessment in studies selected for the systematic review.

Studies Selected	Question and Inclusion	Protocol	Study Design	Comprehensive Search	Study Selection	Data Extraction	Excluded Studies Justification	Included Study Details	Risk of Bias	Funding Sources	Statistical Methods	Risk of Bias in Meta-Analysis	Risk of Bias in Individual Studies	Explanation of Heterogeneity	Publication Bias	Conflict of Interest
Ancan et al., 2020 [14]	Yes	Yes	Yes	Yes	Yes	No	No	No	Yes	N/A	Yes	Yes	Yes	Yes	Yes	Yes
Bolaman et al., 2003 [15]	Yes	Yes	Yes	Yes	Yes	No	No	No	Yes	N/A	Yes	Yes	Yes	Yes	Yes	Yes
Dangour et al., 2011 [16]	Yes	Yes	Yes	Yes	Yes	No	No	No	Yes	N/A	Yes	N/A	Yes	Yes	Yes	Yes
Dangour et al., 2015 [17]	Yes	Yes	Yes	Yes	Yes	No	No	No	Yes	N/A	Yes	Yes	Yes	Yes	Yes	Yes
De Koning et al., 2016 [18]	Yes	Yes	Yes	Yes	Yes	No	No	No	Yes	Yes	Yes	Yes	Yes	Yes	Yes	Yes
Eussen et al., 2006 [19]	Yes	Yes	Yes	Yes	Yes	No	No	No	Yes	Yes	Yes	Yes	Yes	Yes	Yes	Yes
Farvid et al., 2011 [20]	Yes	Yes	Yes	Yes	Yes	No	No	No	Yes	N/A	Yes	Yes	Yes	Yes	Yes	Yes
Fonseca et al., 2013 [21]	Yes	Yes	Yes	Yes	Yes	No	No	No	Yes	N/A	Yes	Yes	Yes	Yes	Yes	Yes
Franques et al., 2019 [22]	Yes	Yes	Yes	Yes	Yes	No	No	No	Yes	N/A	Yes	N/A	Yes	Yes	Yes	Yes
Ghumare et al., 2020 [23]	Yes	Yes	Yes	Yes	Yes	No	No	No	Yes	N/A	Yes		Yes	Yes	Yes	Yes
Janna et al., 2022 [24]	Yes	Yes	Yes	Yes	Yes	No	No	No	Yes	N/A	Yes		Yes	Yes	Yes	Yes
Kanyal et al., 2019 [25]	Yes	Yes	Yes	Yes	Yes	No	No	No	Yes	N/A	Yes	Yes	Yes	Yes	Yes	Yes
Kuzminski et al., 1998 [26]	Yes	Yes	Yes	Yes	Yes	No	No	No	Yes	N/A	Yes	Yes	Yes	Yes	Yes	Yes
Kwok et al., 2019 [27]	Yes	Yes	Yes	Yes	Yes	No	No	No	Yes		Yes	Yes	Yes	Yes	Yes	Yes
Markun et al., 2021 [28]	Yes	Yes	Yes	Yes	Yes	No	No	No	Yes	N/A	Yes	Yes	Yes	Yes	Yes	Yes
Nawaz et al., 2020 [29]	Yes	Yes	Yes	Yes	Yes	No	No	No	Yes	N/A	Yes	Yes	Yes	Yes	Yes	Yes
Serin et al., 2019 [30]	Yes	Yes	Yes	Yes	Yes	No	No	No	Yes	N/A	Yes		Yes	Yes	Yes	Yes

**Table 2:** Description and outcomes as observed in the studies selected for the systematic review.

Author and Year of Study	Sample Size; Mean Age	Study Design	Male:Female Ratio	Study Description/Intervention	Study Outcome/Inference
Ancan et al., 2020 [14]	351 children; 11.8 years	Retrospective study	122:229	The purpose of this study was to assess the clinical response to Vitamin B12 treatment and to report the Vitamin B12 status of patients who were admitted with neurological symptoms. From January 2014 to October 2016, this study focused on children who had Vitamin B12 insufficiency and neurological symptoms. Treatment with intramuscular or oral Vitamin B12 was given to patients whose serum Vitamin B12 levels were less than 300 pg/mL.	To prevent long-term harm, early diagnosis and Vitamin B12 treatment were encouraged. According to the study, those who received Vitamin B12 treatment and had serum Vitamin B12 levels lower than 300 pg/mL experienced a clinical improvement in their neurological symptoms.
Bolaman et al., 2003 [15]	60 patients; ≥16 years	Randomised control trial	61.5:38.5	This was a 90-day prospective, randomised, open-label trial carried out at the Adnan Menderes University Research and Practice Hospital’s Division of Hematology and Department of Internal Medicine (Aydin, Turkey). Patients under the age of 16 with megaloblastic anaemia brought on by cobalamin deficiency were randomised to receive either 1000 g of cobalamin PO or 1000 g of cobalamin IM once day for 10 days (IM group). Both treatments were given after 10 days once a week for 4 weeks, and then once a month going forward. Between days 5 and 10 of treatment, patients were checked for the existence of reticulocytosis until it was found. Hematologic parameters were measured on days 0, 10, 30, and 90, and blood Vitamin B12 concentration was measured on days 0 and 90 to determine the success of the treatment.	Treatment with PO cobalamin was equally efficacious as treatment with IM cobalamin in this trial of patients with megaloblastic anaemia brought on by cobalamin deficiency. In addition, PO medication was less expensive and more tolerated as compared to IM treatment. To ascertain the effectiveness of PO cobalamin treatment, the authors felt that additional long-term studies were required due to the limited sample size and short duration of this investigation.
Dangour et al., 2011 [16]	200 individuals; ≥75 years	Randomised double blind control trial	-	The study’s objective was to determine whether crystalline Vitamin B12 dietary supplements would have improved electrophysiological measures of neurological function in elderly individuals who had biochemical evidence of Vitamin B12 deficiency without anaemia. 200 seniors aged 75 years or over who were randomly assigned to receive either a daily oral tablet containing 1 mg of Vitamin B12 or a corresponding placebo tablet participated in a randomised double-blind placebo-controlled experiment.	Observable improvements in electrophysiological markers of peripheral and central neurosensory responses necessary for movement and sensory function were the main outcome evaluated after 12 months.
Dangour et al., 2015 [17]	201 individuals; 80 years	Randomised control trial	94:107	A double-blind, randomised, placebo-controlled trial was carried out by the authors in South East England’s seven general practices. Participants in the study received 1 mg of crystalline Vitamin B12 or a corresponding placebo as an oral tablet daily for 12 months if they were older than 80 years old and had a mild Vitamin B12 deficiency (serum Vitamin B12 concentrations: 107–210 pmol/L) without anaemia. Prior to and following treatment, peripheral motor and sensory nerve conduction, central motor conduction, a clinical neurologic examination, and cognitive function were evaluated.	A 177% rise in blood Vitamin B12 concentration (641 pmol/L vs. 231 pmol/L), a 331% increase in serum holotranscobalamin (240 vs. 56 pmol/L), and a 17% decrease in serum homocysteine (14.2 vs. 17.1 mol/L) were all linked with allocation to Vitamin B12 when compared to baseline. The trial’s findings did not corroborate the idea that correcting a mild Vitamin B12 deficiency would have positive impacts on later-life neurologic or cognitive function, even in the absence of anaemia and neurologic or cognitive signs or symptoms.
De Koning et al., 2016 [18]	2919 individuals; ≥65 years	Randomised control trial	50:50	This study’s randomised controlled experiment was designed to examine this idea. For two years, participants either received daily doses of Vitamin B12 and folic acid or a placebo. Vitamin D3 was present in both pills. The Geriatric Depression Scale-15 was used to assess depressive symptoms (GDS-15). The SF-12 Mental and Physical component summary scores, the EQ-5D Index score, and the Visual Analogue Scale were used to evaluate health-related quality of life (HR-QoL).	In conclusion, lowering homocysteine concentrations did not reduce depressive symptoms, but it may have had a small positive impact on HR-QoL in older adults with hyperhomocysteinemia who received Vitamin B12 and folic acid supplements for two years.
Eussen et al., 2006 [19]	195 individuals; ≥70 years	Randomised double blind control trial	-	A total of 195 participants in this double-blind, placebo-controlled experiment were randomised to receive Vitamin B12, folic acid, or a placebo for a period of 24 weeks. Methylmalonic acid, total homocysteine (tHcy), and holotranscobalamin (holoTC) concentrations were measured before and after 12 and 24 weeks of treatment to determine the Vitamin B12 status. An thorough neuropsychological test battery that covered the domains of attention, construction, sensomotor speed, memory, and executive function was used to compare cognitive function before and after 24 weeks of treatment.	After receiving treatment, the placebo group’s Vitamin B12 status did not significantly change; however, oral Vitamin B12 supplementation was able to treat mild Vitamin B12 deficiency. Supplementation with Vitamin B12 and folic acid raised red blood cell folate levels and decreased tHcy levels by 36%. The placebo group showed a greater improvement in memory than the group receiving just Vitamin B12. No improvement in other cognitive domains was observed with either Vitamin B12 supplementation alone or in conjunction with folic acid.
Farvid et al., 2011 [20]	67 patients	Randomised double blind control trial	-	Some 75 patients with type 2 diabetes were divided into three treatment groups in this randomised, double-blind, placebo-controlled clinical research. Each group received one of the following daily supplements for four months: Group MVB contains both of the aforementioned mineral and Vitamin supplements along with Vitamin B1 (10 mg), B2 (10 mg), B6 (10 mg), biotin (200 g), B12 (10 g), and folic acid (1 mg); Group P contains a placebo. Group MV contains zinc (20 mg), magnesium (250 mg), Vitamin C (200 mg), and Vitamin E (100 mg).	After 4 months, neuropathic symptoms as measured by the MNSI questionnaire decreased in the group MVB from 3.45 to 0.64, the group MV from 3.96 to 1.0, and the placebo group from 2.54 to 1.95. After four months of supplementation, there was no discernible difference between the three treatment groups on MNSI exams. Patients in the MV and MVB groups did not exhibit any differences in electrophysiological measurements, capillary blood flow, or glycemic management during the course of the 4-month treatment period in comparison to the placebo group.
Fonseca et al., 2013 [21]	214 patients	Randomised double blind control trial		A total of 214 patients with type 2 diabetes and neuropathy (baseline vibration perception threshold [VPT]: 25–45 volts) participated in this multicentre, randomised, double-blind, placebo-controlled trial. Patients were randomised to receive either L-methylfolate calcium 3 mg, methylcobalamin 2 mg, and pyridoxal-5′-phosphate 35 mg for 24 weeks of treatment, or a placebo. The effect on VPT was the main outcome. Other secondary endpoints were plasma levels of folate, Vitamins B(6) and B(12), methylmalonic acid (MMA), homocysteine, and the Neuropathy Total Symptom Score (NTSS-6) and Short Form 36 (SF-36).	In the short term, L-methylfolate, methylcobalamin, and pyridoxal-5′-phosphate (LMF-MC-PLP; brand name Metanx; Pamlab LLC, Covington, La) appears to be a safe and efficient treatment for reducing peripheral neuropathy symptoms. The trial period may have been too brief to demonstrate an effect on VPT, hence the authors argued for more extensive long-term trials.
Franques et al., 2019 [22]	9 patients	Randomised control trial	-	At a hospital, a 3-year retrospective analysis of patients with B12-responsive neuropathy was carried out. The inclusion criteria were electrophysiological research (nerve conduction study) confirmation of neuropathy and improvement of at least 1 point on the overall Overall Neuropathy Limitations Scale following Vitamin B12 therapy.	There were nine patients found. Only four people had low serum B12 levels. Five patients had only sensory neuronopathy, compared to four who had sensorimotor (predominantly sensory) axonal polyneuropathy. Six improved after taking B12 supplements in less than a month.
Ghumare et al., 2020 [23]	100 individuals; 59.32 years	Observational cross-sectional study	61:39	The goal of the current investigation was to determine whether cervical spondylotic myelopathy (CSM) and VB12 insufficiency are related. In our study, 100 CSM patients were examined for VB12 deficiency co-relation. To determine a correlation between VB12 insufficiency and severity of symptoms, demographic data, clinical, radiographic, and laboratory examinations were conducted.	The C5-C6 level (42%) had the highest level of participation, followed by the C4-C5 level (28%) and the C6-C7 level (20%). Motor weakness was noted in 50% of cases, whereas sensory abnormalities were present in 61%. In 47% of instances, VB12 insufficiency was found to be prevalent. 40% of level 1 cord compression cases and 54.1% and 53.8% of level 2 and 3 cord compression cases, respectively, were found to have VB12 deficiencies.
Janna et al., 2022 [24]	331 patients; 63 years	Retrospective cohort study	71:29	Measurements were made of Vitamin B12, methylmalonic acid (MMA), and homocysteine (Hcy) in a retrospective cohort analysis of patients with polyneuropathy. To determine which was most closely associated with Vitamin B12, Hcy or MMA were examined as covariates in linear regression models with Vitamin B12 as the dependent variable. Using logistic regression with elevated metabolites as the dependent variable and Vitamin B12 as the covariate, the threshold Vitamin B12 values for metabolic insufficiency (characterised as elevated metabolites) were established. 42 patients underwent a structured interview to gauge their reaction to Vitamin B12 administration.	B12 was the best Vitamin to link to MMA., and the authors discovered 90% sensitivity at a Vitamin B12 threshold level of 264 pmol/L (358 pg/mL) and 95% sensitivity at 304 pmol/L (412 pg/mL) using increased MMA as a marker for metabolic deficit. Patients reported stabilisation after supplementation in 24% of cases and improvements in 19%. A metabolic or absolute deficit (elevated MMA and Vitamin B12 148 pmol/L) was seen in 88% of patients who improved and 90% of patients who stabilised.
Kanyal et al., 2019 [25]	100 patients; ≥30 years	Cross-sectional study	62:38	This study’s goal was to determine the Vitamin B12 status of T2DM patients using metformin. 100 T2DM patients in total are present at the Dr. Prabhakar Kore Charitable Hospital at KLE. HbA1c and serum Vitamin B12 levels were both calculated.	According to the findings of the current investigation, metformin-induced Vitamin B12 deficiency caused neurologic impairment with symptoms of peripheral neuropathy that may also be classified as diabetic neuropathy. The researchers also found that screening for a subclinical diagnosis of Vitamin B12 insufficiency would be needed at six months or yearly in diabetic patients on metformin therapy.
Kuzminski et al., 1998 [26]	33 patients; 43–92 years	Randomised control trial	2:1	Some 38 patients with recently discovered cobalamin deficiency were randomly randomised to receive cyanocobalamin as either 1 mg intramuscularly on days 1, 3, 7, 10, 14, 21, 30, 60, and 90, or 2 mg orally once day for 120 days. Hematologic and neurologic improvement as well as changes in serum levels of cobalamin (normal, 200 to 900 pg/mL), methylmalonic acid (normal, 73 to 271 nmol/L), and homocysteine (normal, 5.1 to 13.9 mmol/L) were used to assess the efficacy of the treatment.	When treating cobalamin deficiency, 2 mg of cyanocobalamin taken orally once a day was just as beneficial as 1 mg given intramuscularly once a month—and may even be more so.
Kwok et al., 2019 [27]	279 individuals; ≥65 years	Randomised control trial	59.5:40.5	A total of 279 outpatients with moderate cognitive impairment (MCI) who were 65 years old and had serum homocysteine levels of 10.0 mmol/L were randomly randomised to consume two placebo tablets for 24 months or methylcobalamin 500 mg and folic acid 400 mg once daily. Every subject underwent follow-up at intervals of 12 months. The main result was cognitive decline as measured by a rise in the sum of boxes on the clinical dementia rating scale (CDR) (CDR SOB). Global CDR, memory Z score, executive function Z score, and Hamilton Depression Rating Scale (HDRS) score were the secondary outcomes.	Even though the cognitive impairment over the course of two years in the placebo group was minimal, Vitamin B12 and folic acid treatment did not slow cognitive decline in older persons with MCI and increased serum homocysteine. At month 12, the supplement significantly reduced depression symptoms; however, this effect was not long-lasting. The use of aspirin exhibited a detrimental impact on cognitive performance upon interaction with B supplements.
Markun et al., 2021 [28]	16 studies; 66–82 years	Systematic review and meta-analysis	-	This study sought to evaluate the effects of Vitamin B12 alone (B12 alone), along with Vitamin B12 and folic acid with or without Vitamin B6 (B complex), on cognitive function, depressive symptoms, and idiopathic fatigue in patients without advanced neurological disorders or overt Vitamin B12 deficiency.	The clinicians did not discover any proof that supplementing with B12 or B complex had any impact on any subdomain of cognitive function results. Meta-regression also revealed no meaningful correlations between treatment outcomes and any of the putative predictors. Additionally, there was no overall impact of Vitamin supplementation on depression-related indicators. Additionally, because there was just one trial that revealed effects on idiopathic fatigue, no analysis could be done. In patients without advanced neurological problems, Vitamin B12 administration was discovered to be probably useless for enhancing cognitive performance and depressive symptoms.
Nawaz et al., 2020 [29]	-	Literature review	-	This review sought to examine some of the contributing factors to Vitamin B12 (B12) deficiency and its relationship to neurological diseases. The portals that were searched for literature retrieval included PubMed, Google Scholar, the Directory of Open Access Journals (DOAJ), Pak MediNet, and Science Direct.	A review of the literature found that inadequate food intake was the primary cause of this Vitamin’s deficiency, which led to a range of neurological symptoms in both adults and children. Apathy, anorexia, irritability, growth retardation, and developmental regression were among these neurological conditions. Additionally, it could have played a role in the delayed myelination or demyelination of neurons. B12 was found to be an essential micronutrient for young children’s and elders’ healthy brains.
Serin et al., 2019 [30]	38 patients; 0–18 years	Retrospective study	20:18	In view of the characteristics of the patients included, the aim of this study was to stress the significance of early detection of Vitamin B12 deficiency. A total of 38 kids with Vitamin B12 deficiency-related neurological symptoms participated in this retrospective clinical investigation.	After receiving Vitamin B12 supplements, every patient with Vitamin B12 deficiency-related neurological symptoms made a full recovery within a month. In conclusion, anaemia and an increase in mean corpuscular volume are not necessarily connected with the clinical features of Vitamin B12 deficiency, which are general and generic.

## Data Availability

All data are available within the manuscript.
